# New FTY720-docetaxel nanoparticle therapy overcomes FTY720-induced lymphopenia and inhibits metastatic breast tumour growth

**DOI:** 10.1007/s10549-017-4380-8

**Published:** 2017-07-10

**Authors:** Heba Alshaker, Qi Wang, Shyam Srivats, Yimin Chao, Colin Cooper, Dmitri Pchejetski

**Affiliations:** 1 School of Medicine, University of East Anglia, 2.53 BCRE, Norwich Research Park, Norwich, NR47UQ UK; 2 Department of Pharmacology and Biomedical Sciences, Faculty of Pharmacy and Medical Sciences, University of Petra, Amman, Jordan; 3 University of California San Francisco, Health Sciences East 1350, San Francisco, CA 94143-0130 USA; 4 School of Chemistry, University of East Anglia, Norwich, UK

**Keywords:** FTY720, Docetaxel, Sensitisation, PLGA nanoparticle, Breast cancer, Reduced toxicity, Lymphopenia

## Abstract

**Purpose:**

Combining molecular therapies with chemotherapy may offer an improved clinical outcome for chemoresistant tumours. Sphingosine-1-phosphate (S1P) receptor antagonist and sphingosine kinase 1 (SK1) inhibitor FTY720 (FTY) has promising anticancer properties, however, it causes systemic lymphopenia which impairs its use in cancer patients. In this study, we developed a nanoparticle (NP) combining docetaxel (DTX) and FTY for enhanced anticancer effect, targeted tumour delivery and reduced systemic toxicity.

**Methods:**

Docetaxel, FTY and glucosamine were covalently conjugated to poly(lactic-co-glycolic acid) (PLGA). NPs were characterised by dynamic light scattering and electron microscopy. The cellular uptake, cytotoxicity and in vivo antitumor efficacy of CNPs were evaluated.

**Results:**

We show for the first time that in triple negative breast cancer cells FTY provides chemosensitisation to DTX, allowing a four-fold reduction in the effective dose. We have encapsulated both drugs in PLGA complex NPs (CNPs), with narrow size distribution of ~ 100 nm and excellent cancer cell uptake providing sequential, sustained release of FTY and DTX. In triple negative breast cancer cells and mouse breast cancer models, CNPs had similar efficacy to systemic free therapies, but allowed an effective drug dose reduction. Application of CNPs has significantly reversed chemotherapy side effects such as weight loss, liver toxicity and, most notably, lymphopenia.

**Conclusions:**

We show for the first time the DTX chemosensitising effects of FTY in triple negative breast cancer. We further demonstrate that encapsulation of free drugs in CNPs can improve targeting, provide low off-target toxicity and most importantly reduce FTY-induced lymphopenia, offering potential therapeutic use of FTY in clinical cancer treatment.

**Electronic supplementary material:**

The online version of this article (doi:10.1007/s10549-017-4380-8) contains supplementary material, which is available to authorised users.

## Introduction

In the Western world, breast cancer is now the most commonly diagnosed non-cutaneous cancer in women and is the second leading cause of cancer-related deaths [1]. Docetaxel (DTX) is one of the most frequently used chemotherapies for metastatic triple negative breast cancer, however, it has serious adverse effects and many patients relapse on treatment [2]. Thus, any improvement in response to DTX chemotherapy would be of clear benefit. Sphingosine kinase 1 (SK1) is a proto-oncogenic enzyme that is highly expressed in human tumours and has been shown to act as a “cancer signalling hub”. SK1 mediates cancer progression, angiogenesis and cell migration, making it a key molecule in the search for potential anticancer therapies [3]. High levels of SK1 expression were shown in human breast tumours [4–6]. Targeting SK1 in human cancer has therapeutic potential, and improves treatment outcome [3, 7]. FTY720 (FTY) is a sphingosine analogue and a sphingosine-1-phosphate (S1P) receptor antagonist [8]. Pre-clinical studies showed the potential of FTY to downregulate the expression and activity of SK1 and sensitise cancer cells to conventional treatments such as radiotherapy [9], sunitinib [10] and cetuximab [11]. Both downregulation of S1P receptors and SK1 inhibition by FTY lead to downregulation of phosphoinositol-3 kinase-AKT and mitogen activated protein kinase pathways, reduction in cyclin dependent kinases 2/4 and induction of p21 [12, 13]. FTY is currently used for the treatment of multiple sclerosis [14, 15] through induction of immune suppression and lymphopenia, which impairs its use in cancer patients.

Nanoparticle (NP)-based drug delivery systems offer tumour-directed targeting, improved toxicity profiles and capability of co-delivery of drug combinations. NP tumour targeting is based on enhanced permeability and retention effect, whereas tumour vasculature has leaky neo-angiogenic vessels and impaired lymphatic drainage, which allows NP entry and retention within the tumour microenvironment [16]. Additionally, cancer specific peptides or glucosamine [17] linked to NP surface may allow targeting preferentially to the tumour microenvironment [18]. Among different NP based drug vehicles, polymeric NPs have been widely used due to their favourable biodegradability, low antigenicity, and approval for drug use [19]. To date, different strategies have been introduced for preparing polymeric NPs such as physical loading [20] and chemical conjugation [21, 22].

In this study, we have shown for the first time the therapeutic benefit of combining FTY and DTX treatment in triple negative breast cancer. To improve tumour targeting and minimise toxicity, we have designed a combinational NP system where both DTX and FTY were chemically conjugated to poly(lactic-co-glycolic acid) (PLGA). To further improve the efficacy of our nanoformulation, an active cancer targeting ligand, glucosamine [23] was conjugated to PLGA. Using ester and amide covalent bonds provided a sequential delivery of FTY and DTX at the tumour site, increasing their efficacy. In mouse tumours, NP encapsulation reduced systemic toxicity and overcame FTY-induced lymphopenia.

## Materials and methods

### Materials

FTY720 and DTX were purchased from Selleck Chemicals (Houston, USA) and LC laboratories (Woburn, USA), respectively. Caspase-GLO kit was obtained from Promega (Fitchburg, USA). Other reagents and chemicals used were purchased from Sigma-Aldrich (Dorset, UK) unless otherwise specified.

### Synthesis and characterisation of NPs

Detailed NP synthesis method is provided in the supplementary information. Briefly, to conjugate DTX or FTY onto the PLGA polymer, two types of reactions were used. First, the amine group of FTY was protected with di-tert-butyl dicarbonate (t-Boc). It was then conjugated onto the PLGA backbone by steglich esterification. Second, DTX was treated with a solution of 33% trifluoroacetic acid (TFA) to retrieve the amine group. It was then reacted with the carboxyl group of PLGA to form an amide bond. Similar reaction was performed for conjugation of glucosamine onto PLGA. The loading of each drug was quantified by UV spectroscopy. Finally, the DTX-FTY combination PLGA NPs (CNPs) were formulated using an emulsion-solvent evaporation technique with the obtained PLGA-FTY, PLGA-DTX and PLGA-glucosamine. The respective doses of DTX and FTY were 5 nM and 2.5 µM. The size and morphology of CNPs were investigated using scanning electron microscopy (SEM) (JEOL SEM JSM 804A, UK), transmission electron microscope (TEM) (JEOL 2011, UK) and Zetasizer Nano ZS90 (Malvern Instruments, Malvern, UK). The in vitro stability of CNPs was studied by dynamic light scattering (DLS) in five different media: phosphate buffered saline (PBS), Dulbecco’s modified Eagle’s medium (DMEM), DMEM plus 10% heat-inactivated foetal calf serum (FCS) (FirstLink, Birmingham, UK), FCS and 10% human plasma. The Release profile of CNPs was studied in pH 5 PBS using UV–Vis spectrophotometer (Perkin Elmer, UK).

### Cell culture

Human breast cancer cell line MDA-MB-231 and murine breast cancer cell line 4T1 were purchased from ATCC (Manassas, VA, USA), and maintained in DMEM with 10% FCS, 50 U/mL penicillin, 50 μg/mL streptomycin and 2 mM glutamine (Sigma‐Aldrich, St. Louis, MO). Cell lines were kept in culture for up to 30 passages. Cells were seeded to reach 70–80% confluence by the end of the treatment.

### MTT viability assay

Both MDA-MB-231 and 4T1 cells were seeded in 96-well plates and incubated for 24 h. After 24 h starvation, cells were exposed to DTX, FTY, DTX + FTY, empty CNP or CNPs for 48 and 72 h. MTT assay was conducted as previously described [24, 25].

### Caspases activation assay

4T1 and MDA-MB-231 breast cancer cells were cultured in 96 well plates and treated with free individual drugs, their combination, empty CNP or CNPs. After incubation period, 100 µL of caspase3/7-GLO substrate was added and incubated at 37 °C for 1.5 h in the dark. Luminescence was measured thereafter using Pherstart FS (BMG Tech, USA) luminescence reader as described in [24, 25].

### Fluorescent imaging and internalisation of CNPs

To obtain the fluorescent images of CNPs internalisation in breast cancer cells, 4T1 cells were plated on glass coverslips coated with poly-l-lysine. After 24 h, rhodamine tagged NPs were added, followed by lysotracker blue (Life Technologies, UK). Cells were washed with PBS, fixed using 4% paraformaldehyde, mounted onto glass coverslips and imaged using Carl Zeiss confocal microscope. Fluorescence-activated cell sorting (FACS) was also used to confirm and quantify the uptake of CNPs in 4T1 and MDA-MB-231 cells. Cells were incubated with rhodamine tagged CNPs, and collected at each designated time point. They were then stained with 5 µL of Annexin FITC (BD biosciences, UK) and 5 µg/mL aqueous solution of propidium iodide (BD biosciences, UK). The fluorescence signal was measured by FACS (Becton, Dickinson and Company, Erembodegem, Belgium), which gating was constructed based on stained and unstained positive and negative controls.

### Sphingosine kinase 1 (SK1) assay

SK1 assay was performed using radiolabelling as described previously [5, 25, 26], in conditions favouring SK1 activity and inhibiting SK2 activity. Briefly, cell lysates were resuspended in SK1 buffer (20 mM Tris–HCl pH7.4, 20% glycerol, 1 mM 2-mercaptoethanol, 1 mM EDTA, 10 µg/mL Phenylmethanesulfonyl fluoride, 15 mM NaF, 10 µg/mL leupeptin, aprotinine, soybean trypsin inhibitor, 0.5 mM 4-deoxypyridoxine, 40 mM B-glycerophosphate, 1 mM Sodium orthovanadate). Lysates were sonicated and centrifuged at 20,000 g for 30 min at 4 °C. 50 mM sphingosine, 20 mM MgCl_2_, 20 mM ATP and 10 µCi [γ-^32^P]-ATP (6000 Ci/mmol) were added and samples were incubated for 1 h at 37 °C. The reaction was stopped by addition of 1 M HCl, chloroform/methanol/HCl, and 2 M KCL. After centrifugation, the lower organic phase was collected and vaporised. Dried lipids were solubilised with chloroform/methanol (2:1, v/v) and separated by thin layer chromatography on silica gel G60 plates using 1-butanol/ethanol/acetic acid/water (80:20:10:20, v/v) as migration solution. Plates were air-dried, exposed to X-ray film and quantified using Image J software.

### Quantitative real time-PCR (qRT-PCR)

qRT-PCR was performed as described previously [5, 26] using Precision™ 2X qPCR Mastermix with SYBR green^®^ (PrimerDesign Ltd, Southampton, UK). Ct values were exported and analysed using qbase software (Biogazelle NV, Zwijnaarde, Belgium).

### Animal studies

Breast cancer allografts were established in 6–8 week BALB/c nude mice (Charles River Ltd, UK) by injection of 1 × 10^6^ 4T1 cells into their mammary pad. Two weeks after implantation, mice were randomised into treatment groups (*n* = 6/group) and treated twice a week for two weeks with: intravenous tail vein injections of: saline, 5 mg/kg DTX, 3 mg/kg FTY, 5 mg/kg DTX + 3 mg/kg FTY, empty CNPs, CNP1 (5 mg/kg DTX + 3 mg/kg FTY), CNP2 (2 mg/kg DTX + 2 mg/kg FTY). One day after the last treatment, all mice were euthanised and blood was collected. Full blood counts and blood biochemistry were performed by National Veterinary Services Ltd in a blinded manner. Biochemical analysis was performed using a Werfen IL650 photometric clinical chemistry system. Haematological analysis was performed using flow cytometry (Siemens Advia 120). Mice, tumours and individual organs were weighed. 5 × 5 × 5 mm blocks of tumours and individual organs were paraffin fixed and stereomicroscopy was performed using Widefield Zeiss SV11 stereo microscope with emission measured at 530 nm. The levels of fluorescence in each organ were quantified using ImageJ. Animal studies were performed under the Home Office licence and carried out in accordance with the institutional guidelines and regulations for animal welfare and NC3Rs (Replacement, Reduction and Refinement) guidelines.

### Statistical analysis

The statistical significance of differences between the means of two groups was evaluated by unpaired two-sided student’s *t* test. Calculations were performed using GraphPad Prism software. *p* value of <0.05 is considered statistically significant.

## Results

### Synthesis of CNPs

**Fig. 1:**
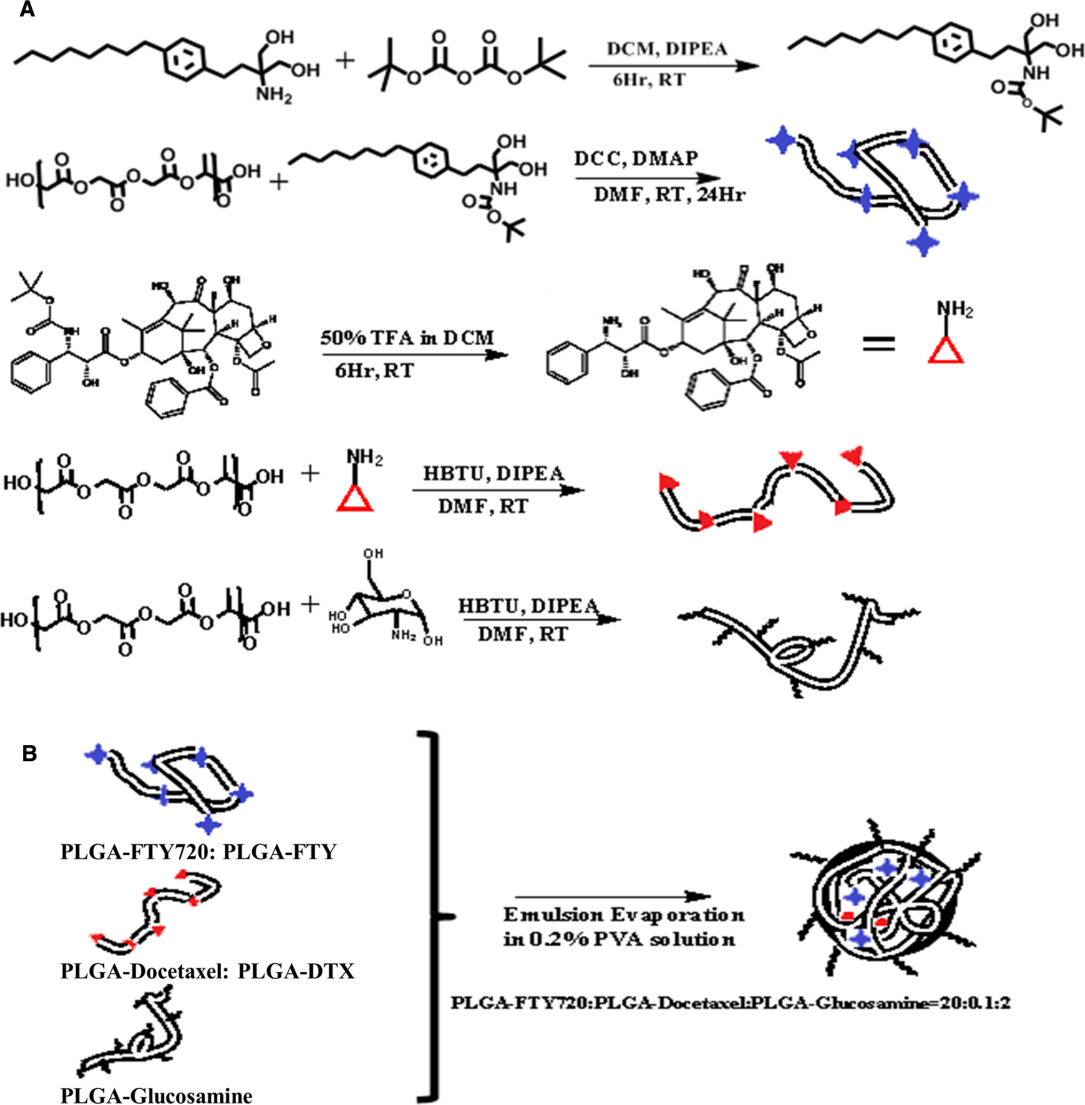
Synthesis of CNPs. **a** Schematic representation of drug modifications: amine head protection in FTY720 (FTY, *blue stars*) to facilitate ester conjugation with PLGA. Deprotection of amine group in docetaxel (DTX, *red triangles*) and amide conjugation with PLGA. Amide conjugation reaction between glucosamine (*black coils*) and PLGA. **b** Synthesis of CNPs through self-assembly from PLGA-FTY, PLGA-DTX and PLGA-glucosamine. *DCC* dicyclo carbodiimide, *DCM* dichloromethane, *DIPEA* diisopropylethylamine, *DMAP* dimethyl amino pyridine, *DMF* dimethyl formamide, *HBTU* N,N,N′,N′-tetramethyl-O-(1H-benzotriazol-1-yl)uronium hexafluorophosphate, *t-Boc* di-tert-butyl dicarbonate, *TFA* trifluoroacetic acid, *PVA* poly vinyl alcohol

CNPs were synthesised using emulsion-solvent evaporation technique using DTX- and FTY-conjugated PLGA (Fig. 1). The amine group along with the octyl tail of FTY enables its recognition as a sphingolipid to inhibit the activity of SK1 (Fig. S1) [27]. Therefore, to design the ester linked PLGA-FTY conjugate, it was essential to protect its amine group using an acid labile moiety as the lysosomal cavity is acidic with a pH 5 [28]. The amine of FTY was protected using t-Boc, an ester anhydride in a basic environment using diisopropylethylamine (DIPEA) (Fig. 1a), which was confirmed using nuclear magnetic resonance (NMR) (Fig. S2). It was then reacted with PLGA wherein the carboxylic acid of the polymer was activated using dicyclo carbodiimide (DCC) and to catalyse ester linkage formation, dimethyl amino pyridine (DMAP) was added (Fig. 1a). In the case of DTX, the functional amine group is protected by t-Boc naturally. Deprotection was carried at 50% TFA in dichloromethane (DCM) (Fig. 1a) to revive the functional group and preserve the anti-mitotic activity of the drug.

The loading of FTY and DTX (assessed using UV–Vis spectrometer) was 60.2 ± 4.5 µg/mg and 30.6 ± 12.5 µg/mg of polymer, respectively. Glucosamine was conjugated to PLGA by an amide bond as shown in Fig. 1a by loading 72.3 ± 2.1 µg per mg of polymer.

The drug ratio in CNPs was designed based on the cell viability testing (Fig. S3), where a combination of 5 nM DTX and 2.5 µM FTY showed potentiating effect in comparison to DTX or FTY alone. Factoring the percentage yield of PLGA-FTY, PLGA-DTX and PLGA-glucosamine conjugates, we designed the combination ratio as 20:0.1:2 (weight %), respectively (Fig. 1b).

### Characterisation of CNPs

**Fig. 2:**
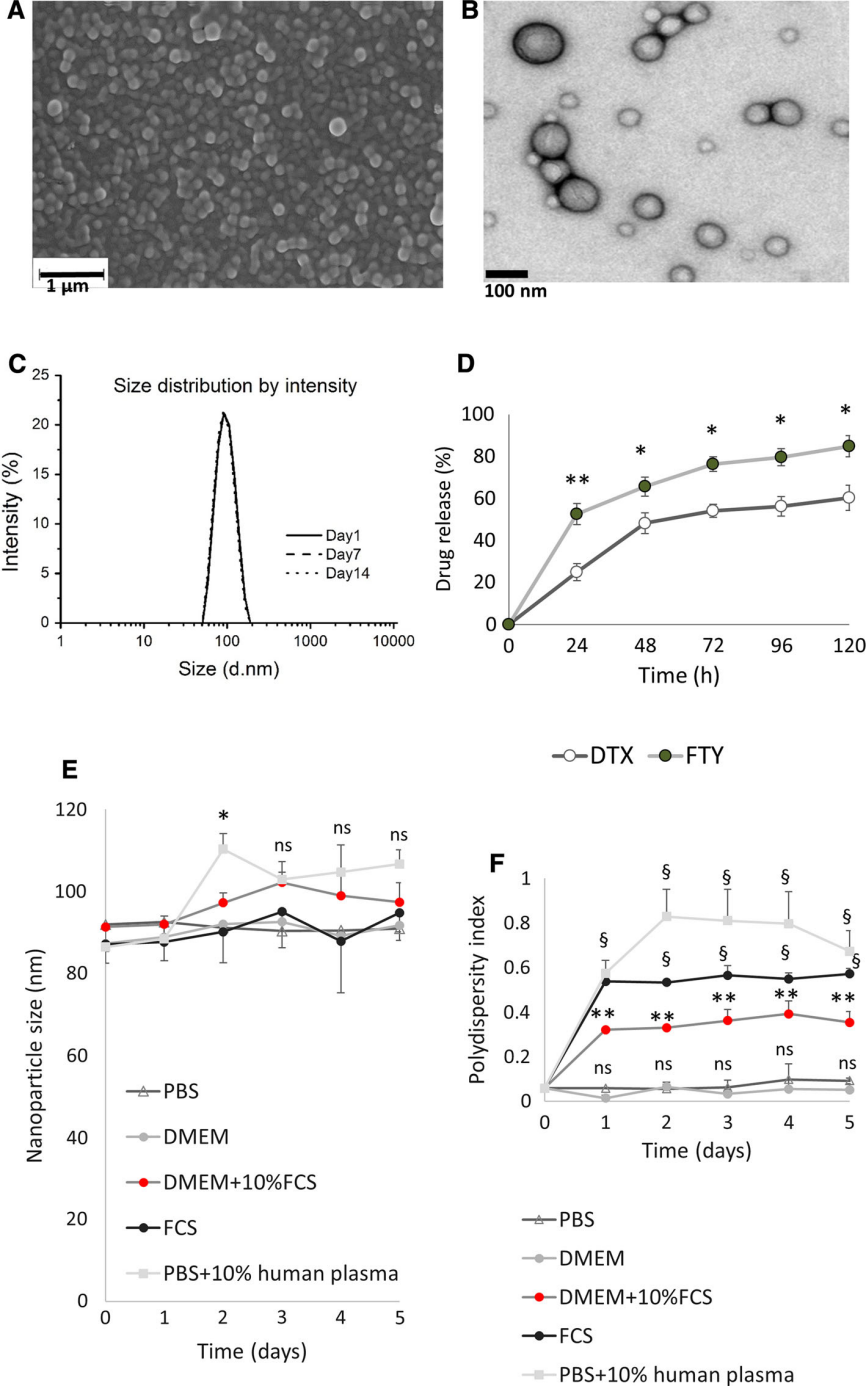
Characterization of CNPs. Morphology of CNPs was assessed by SEM (**a**) and TEM (**b**). *Scale bars* indicate 1 µm and 100 nm, respectively. **c** Distribution of hydrodynamic diameter of PLGA NPs in PBS solution stored for 14 days at 4 °C measured using DLS. **d** Physiochemical release of DTX and FTY was quantified over 120 h using UV–Vis spectrophotometer. **e**, **f** CNPs were incubated in solutions shown on graphs for 5 days. CNP size (**e**) and polydispersity index (PDI) **(f)** were measured as described in methods. Points, mean of three independent experiments performed in triplicate. Data is presented as mean ± SE. *ns* non-significant, *p < 0.05, **p < 0.01, ^§^ *p* < 0.001 (in **d** vs. DTX; in **e**, **f** vs. PBS)

CNPs were characterised by SEM, TEM and DLS as shown in Fig. 2a–c. The CNPs were sphere shaped with the size of 91.51 ± 1.37 nm and a very small polydispersity index (PDI) of 0.05 ± 0.005. These parameters were retained during 14 days. The CNPs were negatively charged with zeta potential of −14.0 ± 0.6 mV.

Sustained release profile is an essential characteristic of NPs as this facilitates reduced drug intake in patients and effective therapy at the diseased site [29]. The release profile of DTX and FTY, was quantified over 120 h in pH 5 acidified PBS (replicating lysosomal environment) (Fig. 2d). Our data show sustained, sequential release of both FTY and DTX from CNPs, with ~52% of FTY and ~25% of DTX released after 24 h (Fig. 2d).

A significant challenge in the application of NPs is to retain their stability in application-associated environments. Aiming at biomedical applications, we evaluated the colloidal stability of CNPs by dissolving them in five different biological media: PBS, DMEM, DMEM with 10% of FCS, pure FCS, and 10% (v/v) human plasma solution (diluted in PBS). The particle characteristics (size and PDI) were measured by DLS over a period of 5 days to monitor if any aggregation would occur. As shown in Fig. 2e, there was no significant change in NP size during the observation period for CNPs suspended in PBS or culture media, suggesting the NPs could be stored for long periods of time with little or no aggregation.

In the presence of serum or human plasma there was no significant change in NP size (Fig. 2e), however there was a significant increase in PDI (Fig. 2f), that was maintained throughout the 5 days of study.

### In vitro internalisation and cytotoxicity effects of CNPs

**Fig. 3:**
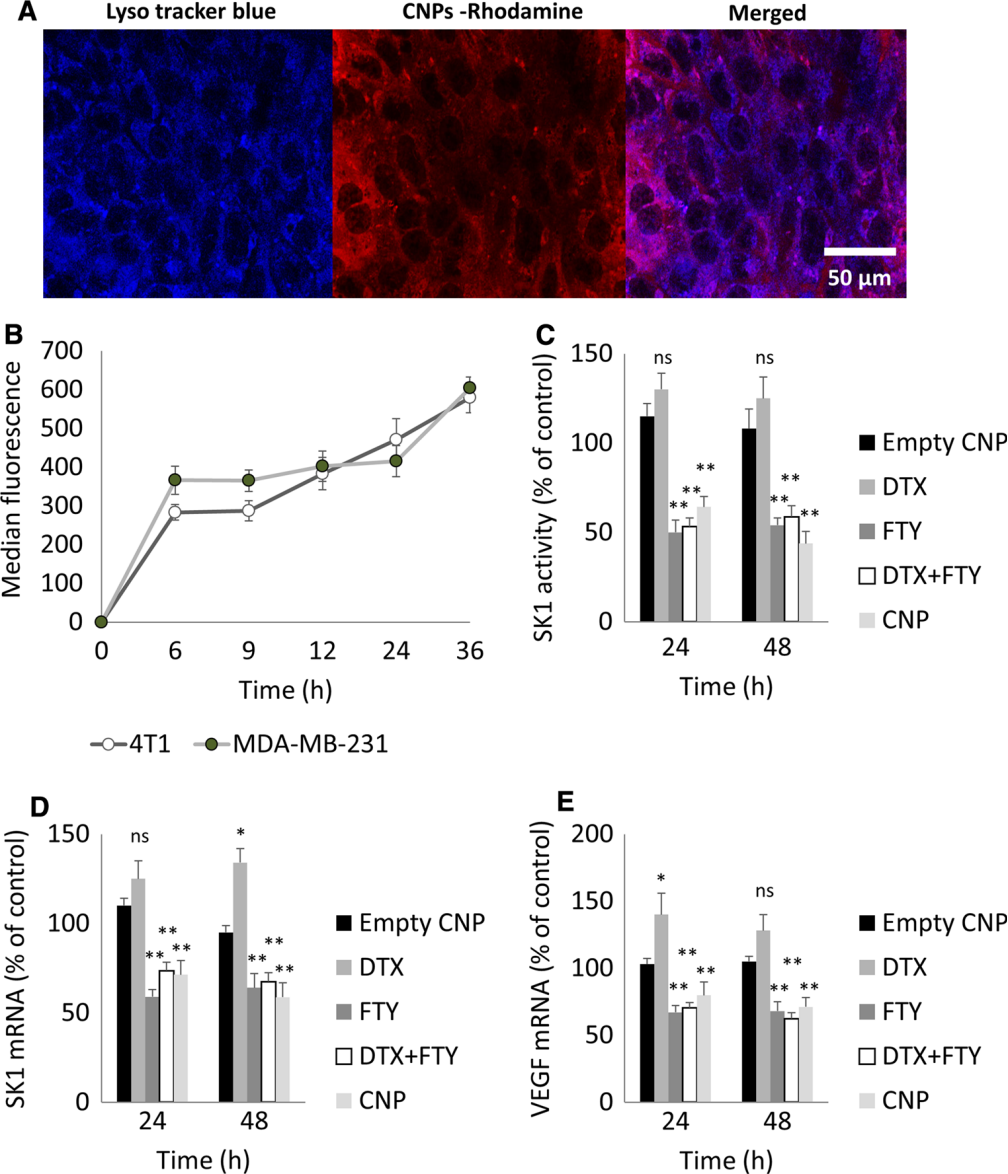
CNPs internalise into cancer cells and reduce SK1 activity and expression. **a** Representative fluorescent microscopy images showing internalisation of CNPs in 4T1 cells. Images were captured at 63X using a Carl Zeiss confocal microscope after 8 h of incubation with rhodamine labelled CNPs. Cells were counter stained with lysotracker blue. Colocalization signals from superimposed images reveal internalisation of CNPs into the endolysosomal compartments. **b** Fluorescence-activated cell sorting (FACS) assisted quantification of rhodamine labelled CNPs internalisation over 36 h time period in MDA-MB-231 and 4T1 cells. **c**–**e** 4T1 cells were treated with DTX-FTY free drug combination (5 nM + 2.5 µM, respectively) or CNPs (containing same doses of DTX and FTY) for 24 h and 48 h. **c** SK1 activity was measured using radiolabeling. Expression of SK1 (**d**) and VEGF (**e**) was determined by qRT-PCR and analysed using qBase software. Graphs show mean of three independent experiments performed in triplicates. Data is presented as mean ± SE. *ns* non-significant, *p < 0.05, **p < 0.01, ^§^ *p* < 0.001 vs. control

4T1 cells were incubated with CNPs labelled with rhodamine 123 for 8 h and then counter stained with lysotracker blue. Fluorescent microscopy revealed internalisation of CNPs into the endolysosomal compartments (Fig. 3a). Flow cytometry showed a time-dependent internalisation of CNPs during 36 h, demonstrating a rapid accumulation of the CNPs within the cells (Fig. 3b).

Following internalisation, in 4T1 cells, CNPs containing 5 nM DTX and 2.5 µM FTY have significantly reduced intracellular SK1 activity. Their effect was inferior to free drugs at 24 h (37 vs. 47% inhibition), but superior at 48 h (56 vs. 42% inhibition) (Fig. 3c). Similar to free drugs, CNPs also reduced SK1 mRNA expression (Fig. 3d). CNPs also had similar effects on expression of vascular endothelial growth factor (VEGF), which was chosen as a surrogate marker of tumour angiogenesis (Fig. 3e). Of note, downregulation of SK1 and VEGF were present in cells treated with FTY alone, while DTX on its own increased SK1 and VEGF mRNA. Similar results were obtained in MDA-MB-231 cells (Figs. S4, S5).

**Fig. 4:**
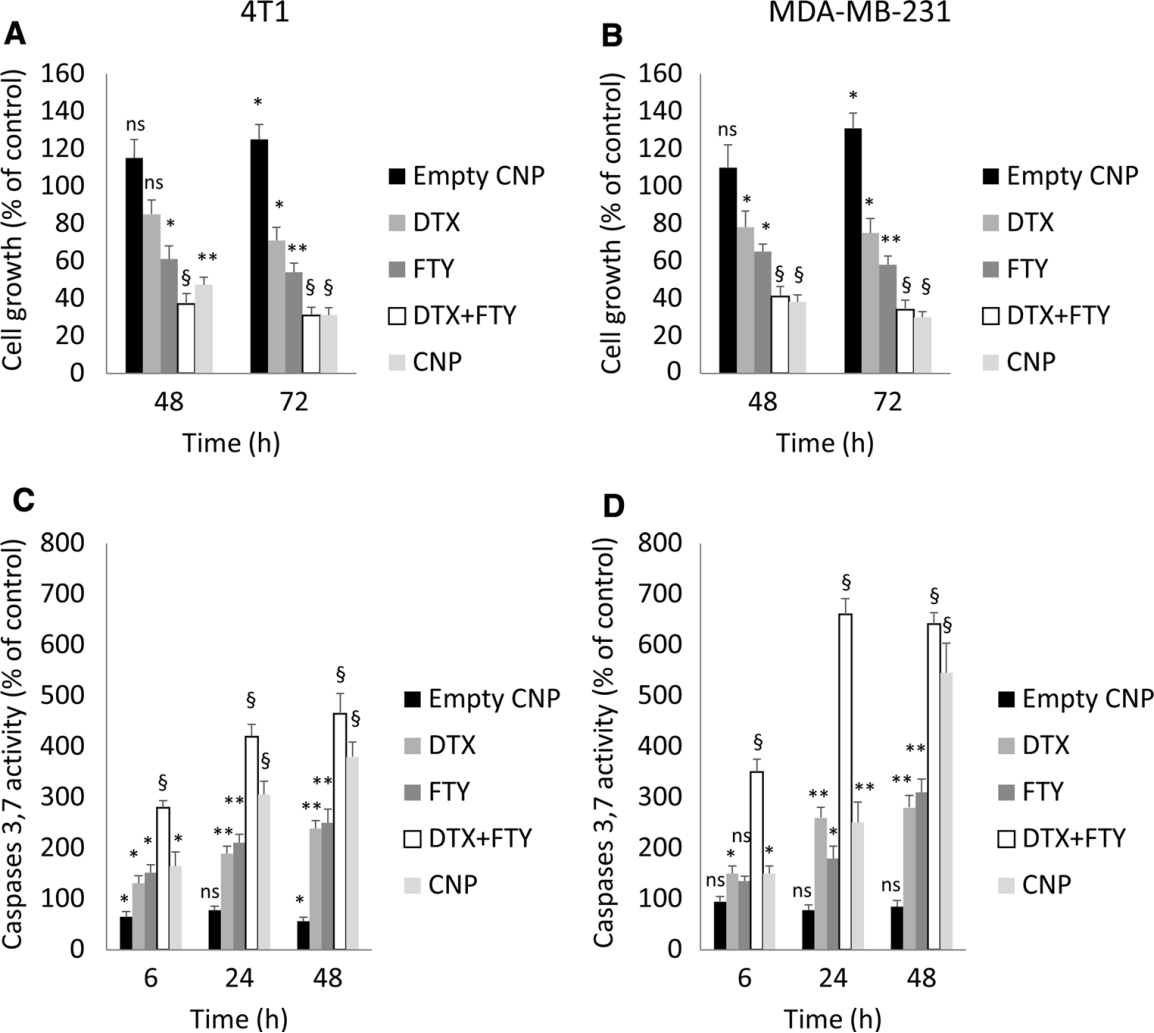
CNPs reduce cancer cell viability and induce apoptosis through caspases 3/7 activation. 4T1 and MDA-MB-231 breast cancer cells were treated with 5 nM DTX + 2.5 µM FTY or CNPs with same doses of drugs for 72 h. Cell viability of 4T1 (**a**) and MDA-MB-231 (**b**) cells was measured using MTT assay. Caspases 3/7 activity in 4T1 (**c**) and MDA-MB-231 (**d**) cells was measured using caspases 3/7 luminescence assay. Graphs depict quantification of three independent experiments performed in triplicates. Data is presented as mean ± SE. *ns* non-significant, *p < 0.05, **p < 0.01, ^§^ *p* < 0.001 vs control

In this study, we demonstrate for the first time that in both triple negative human MDA-MB-231 and mouse 4T1 cell lines free FTY has potentiated the chemotherapy effect of DTX (Figs. 4, S3). This is likely to be achieved through downregulation of SK1 expression and activity (Figs. 3, S3). Similarly to combined free drugs, CNPs containing DTX and FTY have induced significant loss of viability in MDA-MB-231 and 4T1 breast cancer cells (Fig. 4a, b). To study the effect of CNPs on apoptosis induction, caspases 3/7 activity assay was performed. As shown in Fig. 4c, d, both CNPs and free drugs have induced a rapid increase in caspases 3/7 enzymatic activity, which was superior to individual drugs. CNPs had a lag in comparison to free drugs combination, however, showed comparable efficacy at 48-72 h.

### Accumulation of CNPs at tumour sites and their antitumor effect in vivo

**Fig. 5:**
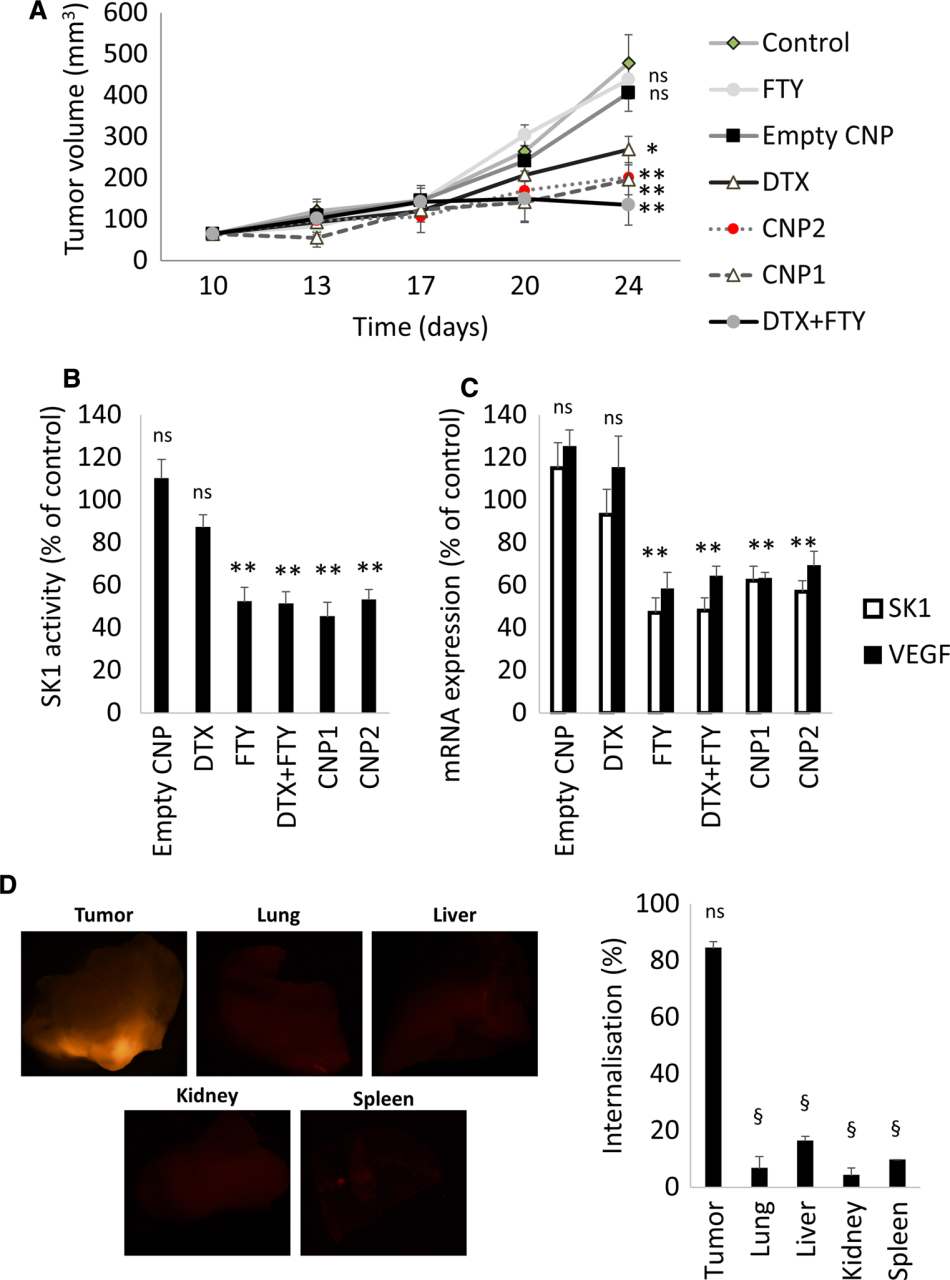
CNPs inhibit 4T1 murine breast tumour growth and SK1 expression and activity. 10^6^ 4T1 breast cancer were implanted subcutaneously in 6–8 week-old female nude mice. Tumours were grown for 2 weeks and then treated for 2 weeks biweekly with intravenous: control (1% dimethyl sulfoxide in saline), empty CNP, 5 mg/kg DTX, 3 mg/kg FTY, free DTX-FTY (5 mg/Kg DTX and 3 mg/Kg FTY), CNP1 (containing 5 mg/Kg DTX + 3 mg/Kg FTY) and CNP2 (containing 2 mg/Kg DTX + 2 mg/Kg FTY). **a** Tumour volume. **b** Tumour SK1 activity. **c** Tumour SK1 and VEGF expression. **d** Fluorescent microscopy of mouse organs and primary tumours. Graph indicates the levels of fluorescence in each organ quantified using ImageJ. Data is presented as mean ± SE. *ns* non-significant, *p < 0.05, **p < 0.01, ^§^ *p* < 0.001 vs. control group

To investigate the in vivo efficacy of CNPs, we inoculated 4T1 cells into the mammary pad of 6–8 week-old female BALB/c nude mice. Tumour-bearing mice were sorted into groups (*n* = 6) and injected with 5 mg/kg DTX, 3 mg/kg FTY, 5 mg/kg DTX + 3 mg/kg FTY, empty CNP, CNP1 (5 mg/kg DTX + 3 mg/kg FTY), CNP2 (2 mg/kg DTX + 2 mg/kg FTY). Two weeks after the inoculation, mice were treated twice a week with intravenous injection of the drugs for two weeks. In the control (saline and the empty CNP) groups, tumours grew progressively and rapidly reaching 478 and 407 mm^3^, respectively (Fig. 5a). Individual FTY did not significantly affect tumour growth with a mean tumour size of 438 mm^3^, while mice in DTX group had tumours of 269 mm^3^. Conversely, the combined free therapy group had a significantly lower mean tumour volume of 135 mm^3^. CNP1 and CNP2 had a similar efficacy in slowing the tumour growth and keeping the tumour volume at ~200 mm^3^ (Fig. 5a). In all treatment groups containing FTY, SK1 activity was ~40% lower than in control group and ~30% lower than in DTX group (Fig. 5b). All treatment groups containing FTY reduced SK1 and VEGF expression by ~40% (Fig. 5c).

To investigate CNPs tumour targeting, rhodamine123 labelled CNPs were injected in the last treatment cycle. Fluorescence imaging of mice organs and primary tumour were obtained using a stereo microscope. As shown in Fig. 5d, the fluorescent signal in tumour was at least four-fold higher than the signal in other organs.

**Fig. 6:**
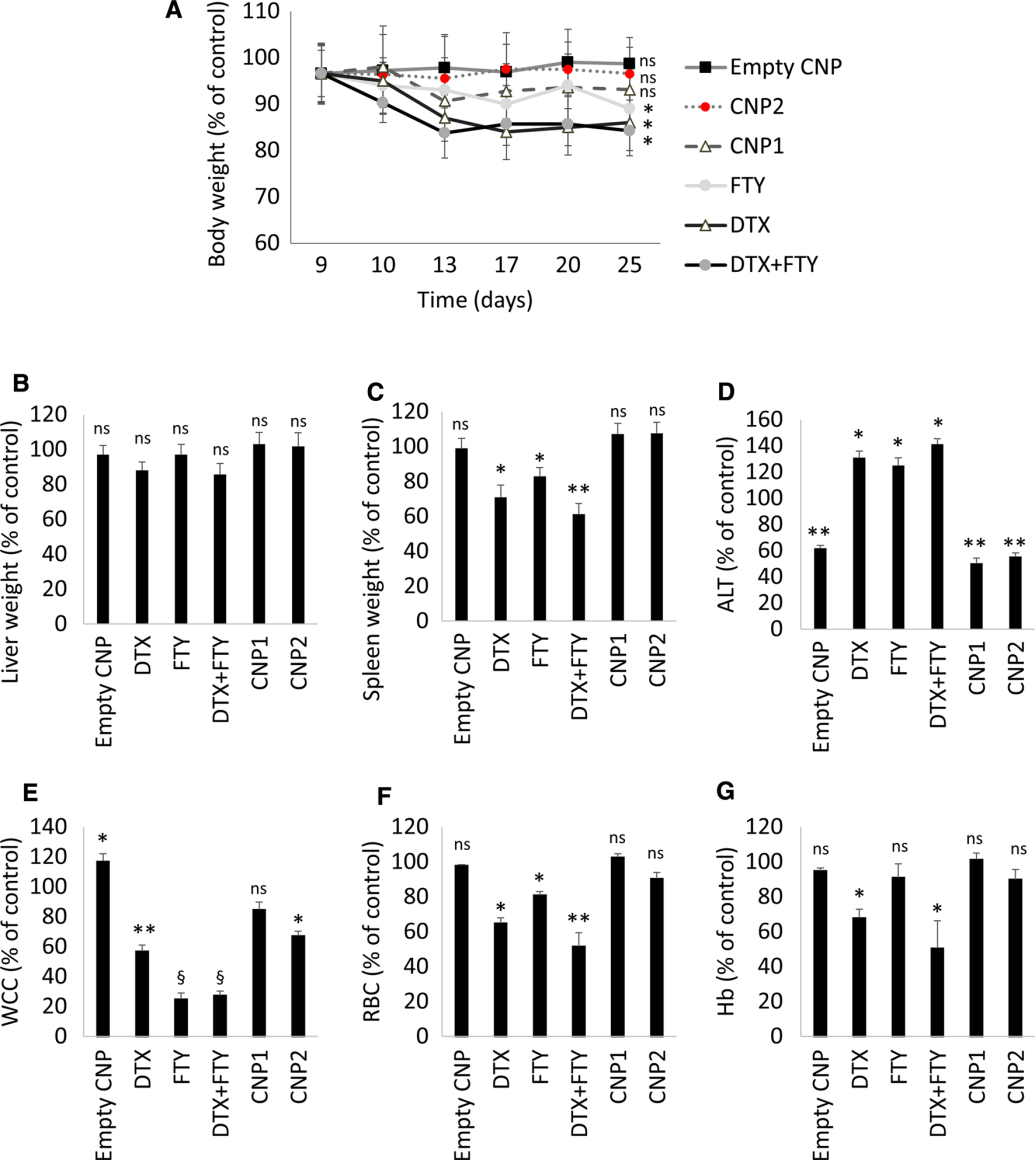
In vivo CNPs demonstrate reduced toxicity in comparison with systemic therapy. 4T1 breast tumours were grown in female nude mice for 2 weeks and treated biweekly in the last two weeks with control, empty CNP, DTX, FTY, free DTX-FTY, CNP1 and CNP2 (as indicated in the legend of Fig. 5). **a** Mouse body weight. **b** Liver weight. **c** Spleen weight. **d** Serum alanine aminotransferase (ALT). **e** White cell count (WCC). **f** Red blood cell count (RBC). **g** Haemoglobin (Hb). Data is normalised vs control group and presented as mean ± SE, *ns* non-significant, *p < 0.05, **p < 0.01, ^§^ *p* < 0.001 vs. control group

Chemotherapy-induced whole body toxicity is a key limiting factor for the administration of effective chemotherapy doses in cancer patients. In mice, systemic free DTX or combined drug therapy, induced a 20% reduction in total body weight (Fig. 6a), insignificantly decreased liver weight (Fig. 6b) and significantly reduced spleen weight (Fig. 6c). Furthermore, free treatments significantly increased liver alanine aminotransferase (ALT) levels and reduced white cell count (WCC), red blood cell count (RBC) and haemoglobin (Hb) (Fig. 6d–g). In contrast, CNPs have markedly reversed all these surrogate markers of overall toxicity and morbidity (Fig. 6).

## Discussion

Enveloping drugs into nanocarriers offers significant advantages including tumour targeting, enhanced delivery and efficacy together with low systemic toxicity. Recent evidence show that nanoformulations provide significant advantage to breast cancer molecular therapies and chemotherapies allowing significant improvement in drug delivery and imaging [30–32]. This is particularly true for docetaxel and paclitaxel [33] where nanoformulations outperform systemic therapies [34, 35]. Abraxane, a 130 nM simple albumin NP-bound paclitaxel that is currently licenced for use in breast, lung and pancreatic cancers. NP binding solubilises hydrophobic paclitaxel and provides a higher maximum whole-blood concentration, shorter time to peak concentration, larger distribution volume and greater clearance than a conventional polyoxyethylated castor oil solubilised paclitaxel [36, 37]. A hallmark phase III trial in women with metastatic breast cancer showed that patients receiving abraxane had longer time to tumour progression (23.0 vs. 16.9 weeks, *p* = 0.006) and objective response rate (33 vs. 19%, *p* = 0.001) than patients receiving conventional paclitaxel [38]. A systematic review and meta-analysis of further randomised clinical trials, showed that the probability of achieving pathological complete response was significantly higher in the abraxane group than in the conventional taxanes group (odds ratio = 1.383, 95% CI 1.141–1.676, *p* = 0.001) [39]. Abraxane was also administered to patients with chemotherapy-naive advanced breast cancer, however it was not superior to paclitaxel and had a trend toward inferiority and higher toxicity [40].

Polymers are highly biocompatible and are commonly used for the synthesis of drug-containing NPs [41]. Polymer NPs show structural stability and are able to encapsulate drugs with high capacity [42]. PLGA is a well-characterised biodegradable polymer widely used for drug delivery and approved for clinical use by the US Food and Drug Administration [29]. It has good toxicity profiles and an enhanced ability to self-assemble into NPs.

We have designed our CNPs aiming for a controlled, time-dependent and sustained drug release profile to achieve maximum therapeutic efficacy [29]. To achieve that DTX was conjugated to the PLGA backbone via an amide bond (harder to degrade in acid environment) and FTY by an ester bond (quicker to degrade) (Fig. 1), leading to a speedier release of FTY (Fig. 2d).

Glucose is an important metabolite for all cells in a living organism as it provides the energy required to carry out all the essential cellular activities. In case of active mitotic cells (such as cancer cells), the requirement of glucose is five-fold higher compared with the normal healthy cells [28]. Here we used glucosamine as a tumour targeting ligand to improve the onsite delivery of CNPs. It was conjugated to PLGA by an amide bond as shown in Fig. 1a.

We aimed the CNPs size to be between 40 and 200 nm to allow the best tumour targeting according to enhanced permeability and retention theory [43]. SEM demonstrated a clear spherical morphology of the CNPs (Fig. 2a), which was shown to enhance their tumour incorporation compared to rod- and disc-shaped NPs [44]. A detailed imaging by TEM further confirmed SEM findings (Fig. 2b). DLS showed a narrow monodisperse CNPs distribution at 91.51 ± 1.37 nm (Fig. 2c). Prolonged incubation of CNPs in PBS showed excellent stability. The CNPs were negatively charged with zeta potential of −14.0 ± 0.6 mV, which in some cases may delay the CNPs cellular uptake due to the negatively charged cell plasma membrane [45].

A significant challenge in the clinical use of NPs is to retain their stability in body environments. The synthesised CNPs had a good colloidal stability in five different biological media: PBS, DMEM, DMEM with 10% of FCS, pure FCS, and 10% (v/v) human plasma solution (diluted in PBS) (Fig. 2e, f). There was no significant change in size and PDI during the observation period for CNPs suspended in PBS or cell culture media, suggesting the NPs could be stored for long periods of time with little or no aggregation. In the presence of serum, an initial of 0.3 and 0.5 increase of PDI was observed and remained unchanged throughout the five days of observation, suggesting some degree of aggregation (Fig. 2f), which however, did not significantly affect average NP size (Fig. 2e). This can be avoided by further modification of the NP surface with polyethylene glycol [46].

Fluorescent microscopy showed a quick and sustained CNPs cellular uptake (Fig. 3a, b). CNPs were colocalised with lysotracker dye demonstrating their preferred endolysosomal distribution. In 4T1 and MDA-MB-231 cells CNPs have successfully supressed SK1 activity (Figs. 3c, S4). They were slightly less efficient than free drugs at 24 h and a little more efficient at 48 h, most likely due to delayed internalisation in comparison to free drugs and longer half-life [47]. Of note, there was no statistical difference between the CNPs and combined free drug treatments. Similar to combined free drugs, CNPs reduced mRNA expression of SK1 by ~30 and ~40% at 24 and 48 h, respectively (Fig. 3d) and to a lesser extent of VEGF (Fig. 3e). All treatments containing FTY have induced a similar downregulation of SK1 and VEGF, while DTX on its own increased SK1 and VEGF mRNA (Figs. 3, S3). These data support the use of FTY as a molecular sensitiser to DTX as it increases the responsiveness of cancer cells to chemotherapy. Similar results were obtained in MDA-MB-231 cells (Fig. S5). Considering a concomitant decrease in SK1 activity and expression, it is possible that the decrease in SK1 activity is at least partially mediated through the downregulation of its expression.

Here we show for the first time that in both 4T1 and MDA-MB-231 triple negative breast cancer cell lines FTY has potentiated the chemotherapy effect of DTX (Figs. 4, S3) and allowed a four-fold reduction in effective DTX dose (from 20 nM as studied previously to 5 nM) [24, 48, 49]. This effect is likely achieved through the downregulation of SK1 (Figs. 3, S3–S5), which we have demonstrated to be a key element in cancer cell resistance to DTX therapy [24, 48, 49]. The distinct conjugation of FTY and DTX via ester and amide bonds, respectively, resulted in speedier FTY release (52 at 24 h vs. 25% for DTX) in pH 5 acidified PBS buffer (replicating lysosomal environment) (Fig. 2d). We hypothesise that this earlier release of FTY may enable lowering cellular defence mechanisms [through downregulation of SK1 activity (Fig. 3)] and allow chemosensitisation to DTX (Figs. S3, 4). Indeed, the effective uptake of CNPs by breast cancer cells has led to a significant loss of cell viability at 48 h and 72 h (Fig. 4a, b). CNPs had comparable cytotoxicity to free drugs, but took 24 h longer to achieve full efficacy, likely due to longer time required for cell uptake and internal degradation in comparison to free drugs. Caspases 3/7 activity assays confirmed that in both breast cancer cell lines, CNPs induced apoptosis through activation of executioner caspases (Fig. 4c, d). Free drugs were more efficient in inducing caspases activation, but had similar levels of cell viability reduction.

To investigate the in vivo efficacy of CNPs, we established subcutaneous breast tumours in female nude mice through inoculation of 4T1 cells into the mammary pad. Tumour-bearing mice were treated biweekly with either DTX, FTY, their combination or CNPs containing equal, or smaller amounts of drugs. Our data clearly show that DTX + FTY is an effective chemotherapy combination for the treatment of triple negative breast cancer tumours, resulting in >3-fold reduction in tumour volume in comparison to control. Importantly, both CNPs (including the ones with lower drug concentrations) had comparable effects to free drugs (Fig. 5a). In all treatment groups containing FTY, SK1 activity and expression was ~40% lower than in control (Fig. 5b, c). These results demonstrate that nanoformulated DTX-FTY has similar antitumor effects to FTY and DTX combination as free drugs. These data point out a possibility of drug dose reduction without losing chemotherapeutic efficacy through effective tumour targeting.

To investigate CNPs targeting properties, rhodamine123 labelled CNPs were injected in the last treatment cycle. Fluorescence signals of mice organs and primary tumour were obtained using a stereo microscope. As shown in Fig. 5d, the fluorescent signal in tumour was four-fold higher than the signal in other organs, suggesting primary tumour targeting and minimal penetration into other tissues.

Chemotherapy-induced toxicity is a key limiting factor for the administration of effective chemotherapy doses in cancer patients. Here we clearly demonstrate that CNPs had significant advantages over the free drugs in all measured parameters including: body weight, liver and spleen size and ALT (Fig. 6). These data suggest that the severe liver damage associated with free drug administration was alleviated through nanoformulation.

The major obstacle for FTY use in cancer patients is significant lymphopenia and anaemia induced by this drug due to T cell sequestration to lymph nodes [8]. Haematological assessment revealed that free FTY has significantly reduced WCC after two weeks of administration (Fig. 6e). In contrast, enveloping drugs in CNPs has improved WCC and RBC by three- and two-fold, respectively, effectively overcoming FTY-induced lymphopenia. Recent data show that low doses of systemic FTY can rather promote tumour growth through accumulation of myeloid-derived suppressor cells, which suppressed antitumor immune response [50]. This important evidence adds a crucial argument towards using targeted FTY tumour delivery for improved outcomes.

Here, we show for the first time that new self-assembled PLGA CNPs containing covalently bound DTX and FTY induce significant apoptosis in triple negative breast cancer cell lines. In accordance with our previous findings in prostate cancer, our data indicate that FTY sensitises cancer cells to DTX, via SK1 inhibition and the CNP platform facilitates this by the sequential release of drugs through conjugation of FTY via a more labile ester bond and DTX via a more stable amide bond. The ~100 nm size range of CNPs and glucosamine presence enable their preferential delivery at the tumour site due to leaky tumour vessels and higher glucose metabolic rate, which is key to reducing systemic side effects. Most importantly, our study provides an evidence that CNPs encapsulation of FTY can reduce systemic lymphopenia and anaemia [9, 51], making it a candidate drug for use in cancer patients.

Overall, we show that using systemic protection, targeted delivery and imaging capability provided by CNPs encapsulation, FTY/DTX combination may have a potential therapeutic use in clinical cancer treatment and demonstrate a platform basis for a SK1 inhibitor/chemotherapy combination in nanoformulation. Future work through peptide or antibody mediated targeting may further enhance the targeting properties and therapeutic outcome.

## Electronic supplementary material

Below is the link to the electronic supplementary material.

Supplementary material 1 (DOC 188 kb)

## Abbreviations

ALT: Alanine aminotransferase
CNPs: Docetaxel and FTY720 combination PLGA nanoparticles
DCC: Dicyclo carbodiimide
DCM: Dichloromethane
DIPEA: Diisopropylethylamine
DLS: Dynamic light scattering
DMAP: Dimethyl amino pyridine
DMF: Dimethyl formamide
DMEM: Dulbecco’s modified Eagle medium
DTX: Docetaxel
FACS: Fluorescence-activated cell sorting
FCS: Foetal calf serum
FTY: FTY720
Hb: Haemoglobin
HBTU: *N,N,N′,N′*-tetramethyl-*O*-(1*H*-benzotriazol-1-yl)uranium hexafluorophosphate
NMR: Nuclear magnetic resonance
NP: Nanoparticle
PBS: Phosphate buffered saline
PDI: Polydispersity index
PLGA: Poly(lactic-co-glycolic acid)
PVA: Poly vinyl alcohol
RBC: Red blood cell count
S1P: Sphingosine-1-phosphate
SE: Standard error of the mean
SEM: Scanning electron microscopy
SK1: Sphingosine kinase 1
t-Boc: Di-tert-butyl dicarbonate
TEM: Transmission electron microscopy
TFA: Trifluoroacetic acid
VEGF: Vascular endothelial growth factor
WCC: White cell count
