# Supplementary material for: New FTY720-docetaxel nanoparticle therapy overcomes FTY720-induced lymphopenia and inhibits metastatic breast tumour growth
Source: Breast Cancer Res Treat. 2017 Jul 10;165(3):531–43. doi: 10.1007/s10549-017-4380-8 (PMC5602005; doi:10.1007/s10549-017-4380-8)
Supplement: Supplementary file 1 — Supplementary material 1 (DOC 188 kb) [file 10549_2017_4380_MOESM1_ESM.doc]

**Additional file**

**New FTY720-Docetaxel nanoparticle therapy overcomes FTY720-induced lymphopenia and inhibits metastatic breast tumour growth**

Heba Alshaker^1,2§^, Qi Wang^1§^, Shyam Srivats^3§^, Yimin Chao^4^, Colin Cooper^1^, Dmitri Pchejetski^1*^

**Detailed drug modification and nanoparticle synthesis procedures**

**Drug modification, characterization and conjugation**

*Synthesis of amine protected FTY720 (FTY)*

FTY (1 eq.) was dissolved in dichloromethane (DCM) and di-tert-butyl dicarbonate (tBoc) (1 eq.) was added to this solution. Diisopropylethylamine (DIPEA) (0.5 eq.) was added and stirred for 6h. The reaction was monitored by thin layer chromatography (TLC) using 98:2 DCM:methanol solvent system. FTY-tBoc has an R_f_ of 0.5 while unreacted FTY remains at the base-line. The reaction was quenched with distilled water. The organic layer was then dried using anhydrous sodium sulfate and DCM was evaporated. The mixture was purified by column chromatography and the product FTY-tBoc having an R_f_ of 0.5 is eluted from the column. This product was then characterized by H^1^ nuclear magnetic resonance (NMR) spectroscopy.

*Synthesis of amine deprotected docetaxel (DTX)*

DTX (1 eq.) was dissolved in a solution of 33% trifluoroacetic acid (TFA) in DCM and was allowed to mix for 6h. The reaction was monitored by TLC wherein amine deprotected DTX does not migrate from the baseline in a 98:2 DCM:methanol solvent system. Unreacted DTX has an R_f_ of 0.6. The reaction is quenched by evaporating both DCM and TFA under pressure.

*Conjugation of FTY to* Poly lactic-glycolic acid (*PLGA)*

Poly lactic-glycolic acid (50:50) (PLGA) (1eq.) was dissolved in dimethyl formamide (DMF). To this, dicyclo carbodiimide (DCC) (0.5 eq.) was added to activate the carboxylic acid of PLGA. To facilitate the ester bond formation, dimethyl amino pyridine (DMAP) (0.5 eq.) was added. FTY-tBoc (1.1 eq.) was then later added to this solution which was stirred over-night. The polymer from this solution was then precipitated using ice-cold diethyl ether. To remove any unreacted FTY-tBoc and other reagents, precipitated polymer was washed with ice-cold ether at least thrice and was dried in a freeze dryer.

*Conjugation of DTX to PLGA*

PLGA (50:50) (1eq.) was dissolved in DMF. To this, ***N,N,N′,N′*-tetramethyl-*O-*(1*H*-benzotriazol-1-yl)uronium hexafluorophosphate (HBTU)** (0.5 eq.) was added to activate the carboxylic acid of PLGA. To facilitate the amide bond formation, DIPEA (0.5 eq.) was added. Amine deprotected DTX (1.1 eq.) from the previous reaction was then later added to this solution which was stirred over-night. The polymer from this solution was then precipitated using ice-cold diethyl ether. To remove any unreacted DTX and other reagents, precipitated polymer was washed with ice-cold ether at least thrice and was dried in a freeze dryer.

*Conjugation of glucosamine to PLGA*

PLGA (50:50) (1 eq.) was dissolved in DMF. To this, **HBTU** (0.5 eq.) was added to activate the carboxylic acid of PLGA. To facilitate the amide bond formation, DIPEA (0.5 eq.) was added. Glucosamine (1.1 eq.) was then later added to this solution which was stirred over-night. The polymer from this solution was then precipitated using ice-cold diethyl ether. To remove any unreacted DTX and other reagents, precipitated polymer was washed with ice-cold ether at least thrice and was dried in a freeze dryer.

*Loading studies*

Concentration based calibration plots were constructed based on UV-Vis spectrophotometer (Perkin Elmer, UK) absorbance of FTY (λ= 266 nm), DTX (λ= 252 nm) and glucosamine (λ= 242 nm) using chloroform as solvent. Later, known quantity of PLGA-FTY, PLGA- DTX, and PLGA-Glucosamine were dissolved in chloroform and the absorbance was compared against the respective calibration curves to calculate the loading of the component/mg of the polymer.

**Nanoformulation and characterisation**

*Nanoformulation*

Nanoparticles (NPs) were formulated using an emulsion-solvent evaporation technique as described: 20 mg PLGA-FTY, 0.1 mg PLGA-DTX, 2 mg PLGA-glucosamine were dissolved completely in 4.5 ml acetone. The entire solution was emulsified into 25 ml 2% aqueous solution of 80% poly vinyl alcohol (PVA) by slow injection with constant homogenization using a tissue homogenizer. This mini-emulsion was added to a 100 ml 0.2% aqueous solution of PVA with rapid mixing for 12h at room temperature to evaporate any residual acetone. NP-size fraction was recovered by ultracentrifugation at 20,000 and 80,000x*g.* The NPs were washed thoroughly with double distilled water to remove excess PVA.

*Characterisation*

Scanning Electron Microscopy (SEM) - The surface morphology of the NPs was studied using SEM (JEOL SEM JSM 804A). A volume of 6 µL of the NP suspension was placed onto an aluminium stub to obtain a uniform layer of particle and dried overnight. Before imaging, the sample was coated with chromium by a sputter gold coater.

Transmission Electron Microscopy (TEM) – The size and shape of the NP was studied using TEM (JEOL 2011). A volume of 6 µL of the NP suspension was applied on holey carbon films on 400 mesh copper grids. After 5 minutes, the suspension was wicked with a filter paper and stained with 1.8% phosphotungstic acid in water (pH 7.2) for 4 minutes after which the staining solution was wicked away using filter paper. The grid was allowed to dry overnight. For dynamic light scattering (DLS) measurement, the size distribution and zeta potential of the NPs was estimated using a Zetasizer Nano ZS90 (Malvern Instruments, Malvern, UK) with 90° optics and a He-Ne Laser.

*Release study*

NPs were suspended in 2 ml of pH 5.5 acidified water (using hydrochloric acid) and were placed in a dialysis bag (MWCO 1000 Da) suspended in 10 ml pH 5.5 water. After stipulated time points 2 ml aliquots of water was extracted and replaced with fresh water (pH 5). The aliquots were freeze dried, dissolved in known quantity of chloroform and the absorbance was measured using UV-Vis spectrophotometer.

**Supplementary figures**


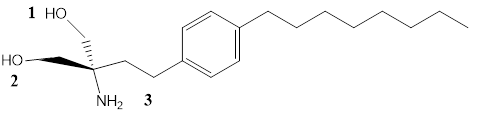


**Figure S1.** **Structure of FTY720**. 1: Pro-(R) Hydroxymethyl mediates lipophilic entrance to target binding site (biologically critical). 2: Pro-(s) Hydroxymethyl is responsible for immunosuppression (chemical and physiochemical importance). 3: 2 amino 4-octylphenyl propane, a sphingolipid moiety for sphingosine kinase recognition and sphingosine-1-phosphate (S1P) receptor binding.

**
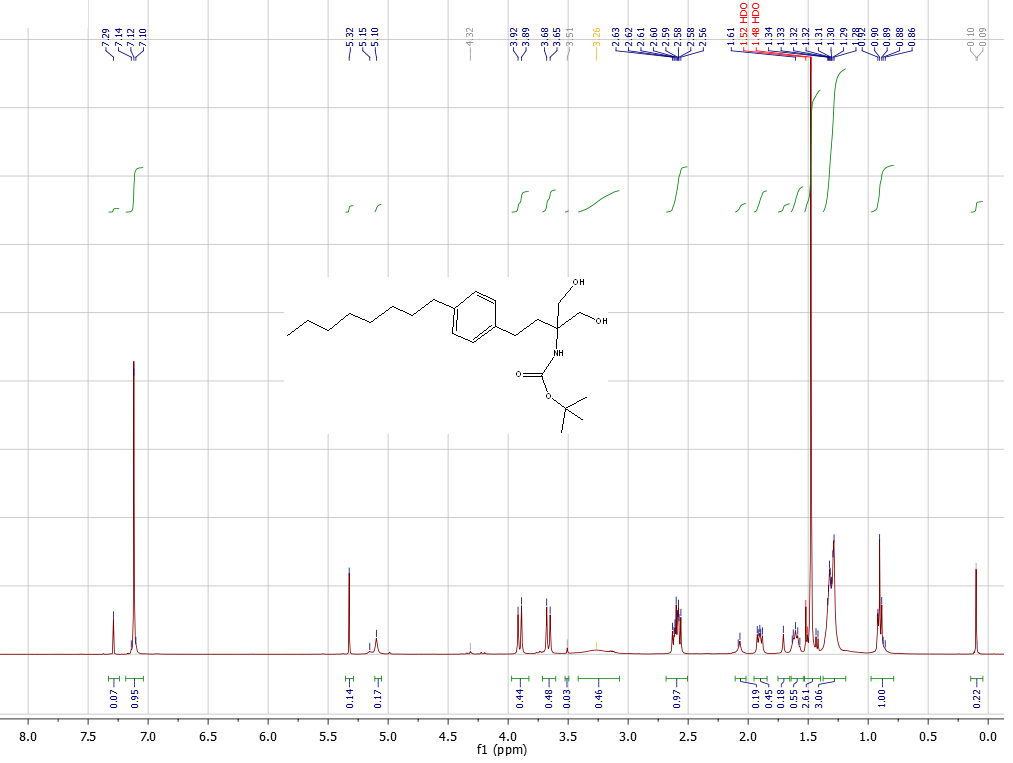
**

**Figure S2**. **Representative nuclear magnetic resonance (NMR) spectroscopy of tBoc protected amine of FTY720 as shown in Figure.1A**.

**
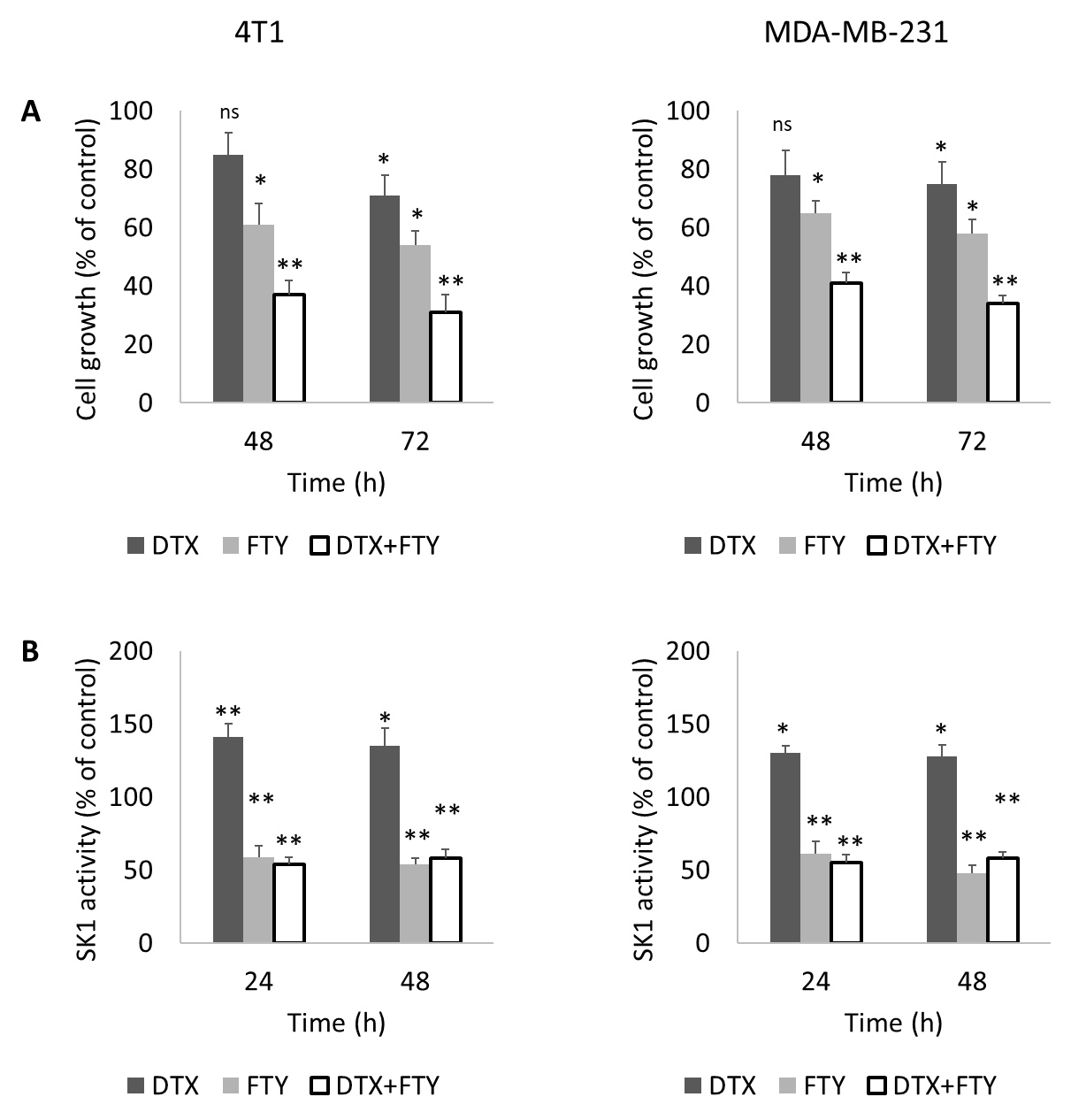
**

**Figure S3**. **Effect of free docetaxel (DTX) and FTY720 (FTY) on breast cancer cell viability and SK1 activity.** 4T1 and MDA-MB-231 cells were treated with 2.5 µM FTY, 5 nM DTX or their combination for indicated times. **A)** Cell proliferation of was measured by MTT assay. **B)** SK1 activity was measured by radiolabelling. Columns, mean of three independent experiments performed in triplicate and normalised to control ± SE. *ns: non-significant, *: p < 0.05, **: p < 0.01 vs control.*

**
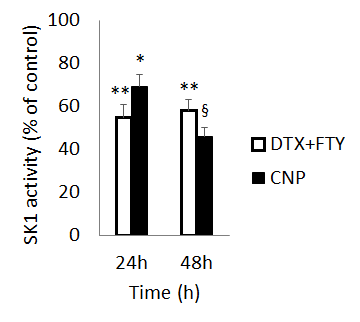
**

**Figure S4**: **CNPs downregulate SK1 activity in MDA-MB-231 cells.** MDA-MB-231 cells were treated with a combination of 2.5 µM FTY and 5 nM DTX or CNPs containing similar amount of drugs for indicated times. SK1 activity was measured by radiolabelling. Columns, mean of three independent experiments performed in triplicate and normalised to control ± SE. *ns: non-significant, *: p < 0.05, **: p < 0.01, §: p<0.001* *vs control.*

**
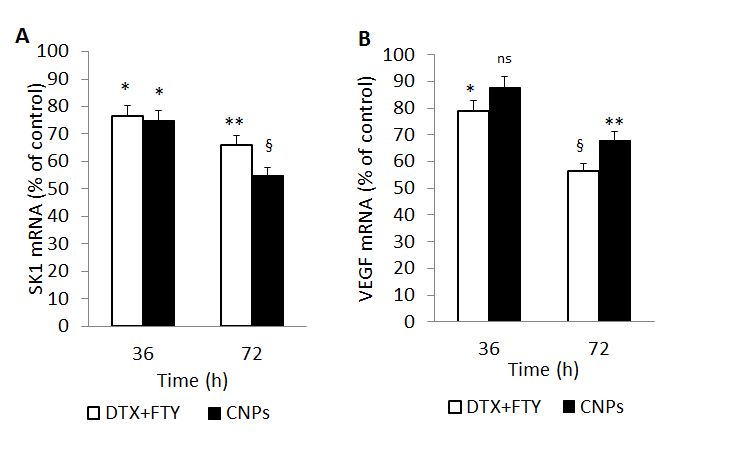
**

**Figure S5**: **CNPs reduce SK1 and VEGF expression in MDA-MB-231 cells.** MDA-MB-231 cells were treated with a combination of 2.5 µM FTY and 5 nM DTX or CNPs containing similar amount of drugs for indicated times. Expression of SK1 **(A)** and VEGF **(B)** was determined by qRT-PCR and analysed using qBase software. Graphs show mean of three independent experiments performed in triplicates. Data is presented as mean and normalised to control ± SE. *ns: non-significant, *: p < 0.05, **: p < 0.01, §: p<0.001* *vs control.*
